# Supplementary material for: A Randomized Controlled Study to Test Front-of-Pack (FOP) Nutrition Labels in the Kingdom of Saudi Arabia
Source: Nutrients. 2023 Jun 27;15(13):2904. doi: 10.3390/nu15132904 (PMC10343722; doi:10.3390/nu15132904)
Supplement: Supplementary file 1 [file nutrients-15-02904-s001.zip › nutrients-2432896-supplementary.pdf]

Table S1: The list of categories and subcategories on NUSMart

ID	Category	Subcategory
1	FRUITS AND VEGETABLES	Fresh fruits
2		Fresh vegetables
3		Frozen fruits
4		Canned fruits
5		Frozen vegetables
6		Canned vegetables
7	SEAFOOD, MEAT, AND POULTRY	Fresh fish and seafood
8		Fresh meat
9		Frozen fish and seafood
10		Frozen meat
11		Fresh Poultry
12		Frozen Poultry
13	STAPLES	Accompaniments
14		Noodles
15		Pasta
16		Rice
17		Legumes, Lentils, Stuffing and Sides
18	DAIRY AND EGGS	Egg and egg products
19		Butter and margarine spreads
20		Cheese spreads and labneh
21		Fresh cheese
22		Cream, creamer and condensed milk
23		Yoghurt
24		Cheese slices, blocks and cubes
25		Shredded cheese
26	BREAKFAST CEREALS	Hot Cereals
27		Cold Cereals
28	BAKERY	Baking ingredients and mixes
29		Bread, buns and bread products
30		Cakes, pastries and sweet goods
31	PROCESSED SEAFOOD, MEAT, AND POULTRY	Fish products
32		Meat products
33		Poultry products
34	OILS, CONDIMENTS AND DRESSINGS	Dressings and vinegar
35		Mayonnaise
36		Oils and ghee
37	SAUCES AND SEASONINGS	Cooking sauces
38		Spices & Seasoning
39		Pasta Sauces
40		Pickled Condiments
41		Stocks
42		Table Sauces
43	SNACKS	Crisps and Chips
44		Biscuits, Crackers, Cookies and Rusk
45		Dried Fruit
46		Frozen Snacks
47		Popcorn
48		Snack Mixes
49		Cereal/ Energy bars
50	LEGUMES, NUTS AND SEEDS	Legumes, Nuts and Seeds
51	SOUP	Dry soup
52		Wet Soup
53		Caramel and Cream spreads
54		Chocolate spreads

55	SPREADS	Dips
56		Honey
57		Jam and Marmalade
58		Nut spreads
59		Savoury spreads
60	CONVENIENCE MEALS	Instant noodles
61		Instant porridge
62		Pizza
63		Prepared meals
64	DAIRY DRINKS	Fresh milk
65		Flavoured milk
66		Cultured milk drinks, yoghurt drinks and laban
67		Soy, nut and grain-based milk
68	HOT BEVERAGES	Coffee
69		Tea
70		Malt and other hot beverages
71	READY-TO-DRINK	Carbonated Soft Drinks
72		RTD (Iced) Coffee
73		RTD (Iced) Tea
74		Other RTDs
75		Fruit Juices
76		Cordials and Nectar
77	SPORTS AND ENERGY DRINKS	Energy Drinks
78		Sports and Isotonic Drinks
79	DESSERTS AND ICE CREAM	Cheesecakes
80		Ice Cream
81		Jellies and Puddings
82		Halawa and Tahina
83		Sweet sauces and syrups
84		Compotes
85	CHOCOLATE CONFECTIONERY	Chocolate Bars
86		Chocolate blocks
87		Chocolate packs and pouches
88		Chocolate boxes
89	SUGAR AND GUM CONFECTIONERY	Chewy sweets and marshmallows
90		Lollipops and hard candy
91		Mint and lozenges
92		Pastilles, gums, jellies and chews
93		Toffees, caramels and nougat
94	SWEETENERS AND SUGAR	Artificial sweeteners
95		Natural sweeteners
96		Sugar
97		Sugar cubes and crystals
98	VEGETARIAN MEAT AND SEAFOOD	Plant-based meat and seafood alternatives

Table S2 Descriptive statistics by study completion status

	(1) Dropout sample Mean [SE]	(2) Analysis sample Mean [SE]	p-value (1)-(2)
Age	31.047	31.694	0.100
	[0.290]	[0.266]	
Female=1	0.468	0.488	0.456
	[0.018]	[0.020]	
Household Size	4.902	4.697	0.075
	[0.084]	[0.079]	
University degree and above	0.722	0.819	0.000***
	[0.016]	[0.015]	
Income 15,000SAR and above	0.308	0.450	0.000***
	[0.017]	[0.019]	
No disease=1	0.267	0.407	0.000***
	[0.016]	[0.019]	
Observations	765	656	

Note: ** p<0.05, *** p<0.01

Table S3 Dropout rates by study arm

	Full sample	Arm 1	Arm 2	Arm 3	p-value (1)-(2)	p-value (2)-(3)	p-value (1)-(3)
Dropout rate	0.538	0.549	0.514	0.553	0.283	0.226	0.891
	[0.013]	[0.023]	[0.023]	[0.023]			
Observations	1421	474	475	472			

Table S4 Effects of the NS and WL on the weighted average NS point and per-serving energy and nutrients (foods and beverages)

	weighted NS point per serving	kcal per serving	sugar per serving
NS arm	2.53***	-29.5	-2.10***
	[1.67,3.39]	[-164.2,105.1]	[-2.85,-1.35]
WL arm	0.63	-98.2*	-0.48
	[-0.20,1.47]	[-205.6,9.15]	[-1.27,0.31]
Age	0.070**	1.98	-0.044*
	[0.017,0.12]	[-3.60,7.56]	[-0.092,0.0038]
Female	0.73*	59.9	-1.11***
	[-0.0050,1.46]	[-35.3,155.0]	[-1.75,-0.47]
Household Size	-0.12	-1.58	-0.0071
	[-0.27,0.037]	[-18.3,15.2]	[-0.16,0.14]
University degree and above	0.040	76.0*	-0.19
	[-0.91,0.99]	[-9.77,161.7]	[-1.17,0.78]
Income 15,000SAR and above	0.15	40.8	-0.0047
	[-0.58,0.88]	[-65.2,146.9]	[-0.68,0.67]
taste Best=1	0.86	69.7	-1.92**
	[-0.69,2.40]	[-128.4,267.7]	[-3.59,-0.25]

price Best=1	1.55*	33.8	-2.83***
	[-0.097,3.20]	[-166.4,234.0]	[-4.55,-1.10]
health Best=1	2.18***	-70.4	-2.58***
	[0.57,3.80]	[-259.9,119.2]	[-4.22,-0.93]
variety Best=1	1.89**	-35.7	-2.48***
	[0.25,3.52]	[-241.1,169.6]	[-4.27,-0.68]
No disease=1	0.68*	86.5	-0.64**
	[-0.047,1.41]	[-24.7,197.7]	[-1.26,-0.019]
Constant	28.4***	74.9	12.4***
	[25.8,31.1]	[-188.0,337.8]	[9.84,15.0]
Observations	656	656	656
R^2	0.099	0.024	0.104

	sodium per serving	Saturated fat per serving	Total kcal
NS arm	-120.1	6.13	-31647.1
	[-604.1,363.9]	[-25.1,37.4]	[-92281.1,28986.8]
WL arm	28.8	-24.6**	-40778.0
	[-713.9,771.4]	[-47.7,-1.44]	[-94524.9,12968.8]
Age	-3.08	-2.04	750.5
	[-41.3,35.1]	[-5.28,1.20]	[-2562.2,4063.2]
Female	-425.1	-14.6	26704.9
	[-1012.6,162.5]	[-47.1,17.8]	[-17086.3,70496.1]
Household Size	113.1	-4.08	26640.8***
	[-40.4,266.5]	[-9.99,1.83]	[8473.2,44808.5]
University degree and above	-404.1	3.53	-6416.8
	[-1191.9,383.6]	[-26.0,33.0]	[-63910.3,51076.7]
Income 15,000SAR and above	186.0	31.3*	34872.3
	[-311.8,683.8]	[-2.56,65.2]	[-13252.8,82997.4]
taste Best=1	155.9	-2.39	74421.7*
	[-581.9,893.8]	[-34.0,29.3]	[-4333.3,153176.6]
price Best=1	169.2	15.7	48203.7
	[-662.8,1001.3]	[-24.7,56.1]	[-39807.8,136215.2]
health Best=1	3.12	17.9	-13141.1
	[-748.7,754.9]	[-37.4,73.1]	[-75789.6,49507.4]
variety Best=1	1406.2	15.7	-811.5
	[-699.4,3511.8]	[-32.0,63.4]	[-72790.3,71167.3]

No disease=1	-485.3**	-0.094	7470.3
	[-927.3,-43.3]	[-29.7,29.5]	[-36963.3,51903.8]
Constant	754.7	104.4*	-58601.6
	[-1202.7,2712.2]	[-12.6,221.5]	[-170920.3,53717.1]
Observations	656	656	656
R^2	0.029	0.023	0.054

	Total sugar	Total sodium	Total saturated fat
NS arm	-1438.7***	-373271.7*	-7218.7
	[-2090.1,-787.3]	[-761041.8,14498.4]	[-25241.0,10803.6]
WL arm	-404.4	-110717.3	-17205.9**
	[-1158.6,349.8]	[-674611.1,453176.6]	[-33249.0,-1162.9]
Age	-12.8	5764.0	-530.7
	[-46.3,20.7]	[-24064.8,35592.8]	[-1671.0,609.5]
Female	-615.9**	-183076.1	-8035.1
	[-1189.7,-42.0]	[-664583.6,298431.4]	[-22425.9,6355.6]
Household Size	749.5***	191485.7**	2306.1
	[616.7,882.3]	[6081.1,376890.2]	[-2588.0,7200.3]
University degree and above	-147.3	-246372.0	-9583.6
	[-868.3,573.6]	[-963414.6,470670.6]	[-31246.4,12079.3]
Income 15,000SAR and above	-198.0	155707.1	12073.9*
	[-742.6,346.6]	[-243453.5,554867.7]	[-946.7,25094.4]
taste Best=1	-484.0	192075.8	-2384.2
	[-1651.5,683.4]	[-561668.3,945819.9]	[-27718.4,22949.9]
price Best=1	-1302.3**	12446.7	-867.2
	[-2507.8,-96.8]	[-706232.9,731126.2]	[-26531.2,24796.9]
health Best=1	-1301.1**	-74975.3	1572.9
	[-2449.9,-152.4]	[-803584.6,653633.9]	[-26458.4,29604.2]
variety Best=1	-881.5	650809.3	847.8
	[-2219.2,456.2]	[-785200.0,2086818.6]	[-29424.2,31119.8]
No disease=1	-281.5	-381164.3**	-3822.8
	[-803.0,240.1]	[-683689.4,-78639.1]	[-16526.0,8880.3]
Constant	2763.1***	-149321.5	40005.1*
	[1106.7,4419.5]	[-1488676.6,1190033.6]	[-6359.5,86369.7]
Observations	656	656	656
R^2	0.205	0.044	0.020

Notes: 95% confidence intervals are in brackets. * p<0.1, ** p<0.05, *** p<0.01

Table S5 Effects of the NS and WL on weighted average NS point and per-serving energy and nutrients (beverages only)

	weighted NS point per serving	kcal per serving	sugar per serving
NS arm	2.51***	-24.5***	-6.31***
	[1.29,3.73]	[-36.7,-12.3]	[-10.1,-2.49]
WL arm	1.63***	-17.9***	-5.02***
	[0.47,2.79]	[-29.8,-5.95]	[-8.36,-1.69]
Age	0.030	0.0012	0.076
	[-0.042,0.10]	[-0.70,0.70]	[-0.14,0.29]
Female	1.11**	-8.01	-3.61**
	[0.11,2.11]	[-17.9,1.84]	[-6.58,-0.64]
Household Size	0.055	0.18	0.31
	[-0.17,0.28]	[-1.74,2.09]	[-0.28,0.91]
University degree and above	0.10	-3.65	-0.068
	[-1.31,1.51]	[-15.9,8.58]	[-4.18,4.04]
Income 15,000SAR and above	-0.17	5.20	1.37
	[-1.24,0.89]	[-5.24,15.6]	[-1.97,4.72]
taste Best=1	1.08	-9.58	-6.02
	[-1.30,3.45]	[-33.1,14.0]	[-13.7,1.62]
price Best=1	1.92	-23.7*	-9.48**
	[-0.64,4.48]	[-48.1,0.69]	[-17.1,-1.87]
health Best=1	1.83	-18.5	-8.72**
	[-0.67,4.34]	[-43.6,6.55]	[-16.5,-0.96]
variety Best=1	1.10	-15.2	-4.50
	[-1.45,3.65]	[-39.8,9.39]	[-13.0,4.05]
No disease=1	-0.31	-1.25	1.31
	[-1.32,0.70]	[-11.6,9.11]	[-1.60,4.23]
Constant	26.0***	129.2***	23.5***
	[22.3,29.7]	[93.9,164.5]	[13.2,33.8]
Observations	525	525	525
R^2	0.053	0.056	0.067

	sodium per serving	Saturated fat per serving	Total kcal
NS arm	-3.81	-0.028	-1326.8
	[-13.8,6.19]	[-0.29,0.24]	[-3246.9,593.3]
WL arm	-2.65	-0.23*	-1217.2

	[-13.2,7.92]	[-0.47,0.017]	[-3380.4,946.1]
Age	-0.34	-0.018**	-82.7
	[-0.98,0.30]	[-0.034,-0.0024]	[-193.9,28.5]
Female	-2.03	-0.041	-2456.6***
	[-10.9,6.82]	[-0.25,0.17]	[-4171.4,-741.8]
Household Size	-1.76	-0.013	798.4***
	[-3.91,0.40]	[-0.062,0.036]	[358.6,1238.2]
University degree and above	-2.61	-0.041	470.9
	[-15.1,9.93]	[-0.34,0.26]	[-1833.5,2775.4]
Income 15,000SAR and above	-2.35	-0.0057	-800.2
	[-11.4,6.73]	[-0.22,0.20]	[-2603.8,1003.4]
taste Best=1	7.11	0.23	-5988.0**
	[-12.2,26.4]	[-0.12,0.59]	[-10616.5,-1359.6]
price Best=1	6.52	0.26	-8982.7***
	[-14.3,27.4]	[-0.14,0.65]	[-13534.2,-4431.2]
health Best=1	6.56	0.37*	-7968.8***
	[-13.1,26.2]	[-0.029,0.77]	[-12534.9,-3402.7]
variety Best=1	6.09	0.22	-6621.4***
	[-14.7,26.9]	[-0.19,0.62]	[-11554.0,-1688.8]
No disease=1	3.64	-0.14	-804.2
	[-5.43,12.7]	[-0.36,0.079]	[-2387.1,778.6]
Constant	78.8***	1.53***	13412.3***
	[45.7,111.9]	[0.79,2.26]	[7392.5,19432.2]
Observations	525	525	525
R^2	0.016	0.023	0.100

	Total sugar	Total sodium	Total saturated fat
NS arm	-298.4	-601.5	-14.9
	[-674.0,77.3]	[-1930.4,727.5]	[-35.1,5.28]
WL arm	-304.2	-513.8	-9.96
	[-674.1,65.6]	[-2012.9,985.3]	[-35.6,15.6]
Age	-8.11	-82.7**	-1.17*
	[-29.1,12.9]	[-164.4,-0.97]	[-2.40,0.063]
Female	-533.6***	-1138.5*	-5.18
	[-843.7,-223.5]	[-2321.9,44.8]	[-23.6,13.3]
Household Size	162.2***	428.8**	3.31

	[73.9,250.5]	[91.8,765.7]	[-0.82,7.44]
University degree and above	76.2	237.7	7.69
	[-349.3,501.8]	[-1519.0,1994.3]	[-18.5,33.9]
Income 15,000SAR and above	-122.3	-1029.6	-12.2
	[-446.5,202.0]	[-2271.3,212.0]	[-32.9,8.56]
taste Best=1	-958.8***	-3037.5	-38.7
	[-1646.3,-271.3]	[-7086.8,1011.8]	[-100.8,23.4]
price Best=1	-1497.5***	-4940.3**	-64.3**
	[-2181.0,-814.0]	[-8983.2,-897.5]	[-126.0,-2.53]
health Best=1	-1387.2***	-3969.6**	-48.1
	[-2061.2,-713.2]	[-7928.4,-10.8]	[-108.5,12.4]
variety Best=1	-872.2**	-3472.5*	-51.6*
	[-1719.8,-24.6]	[-7576.1,631.1]	[-112.7,9.58]
No disease=1	-14.6	-178.5	-16.2*
	[-310.8,281.7]	[-1296.3,939.3]	[-33.2,0.69]
Constant	1983.4***	8177.6***	121.6***
	[1004.3,2962.5]	[2757.4,13597.8]	[38.4,204.8]
Observations	525	525	525
R^2	0.112	0.063	0.041

Notes: 95% confidence intervals are in brackets. * p<0.1, ** p<0.05, *** p<0.01

Table S6 Post-Study Survey Results

	Control	Warning	NS
**1. NS Introductory video**			
1-1. The percentage of the NS arm participants who reported to watch the video.	N/A	N/A	86%
1-2. The percentage of the participants who watched the video (1.1) correctly answered the question asking what the video is about.	N/A	N/A	56%
**2. FOP labels**			
2-1. The percentage of participants who chose the correct label design they recalled seeing in their shop.	NA	73%	78%
2-2. The percentage of participants who watched the video (1.1.) correctly answered the question asking if NS D label best applies to either Coca-Cola classic or a fresh apple.	NA	NA	90%
